# Intrauterine device management by nurses in Primary Health Care

**DOI:** 10.1590/0034-7167-2024-0340

**Published:** 2025-06-13

**Authors:** Eliane Kelly Ribeiro da Silva, Helisamara Mota Guedes, Débora Dupas Gonçalves do Nascimento, Albert Schiaveto de Souza, Sebastião Junior Henrique Duarte

**Affiliations:** I Fundação Oswaldo Cruz. Campo Grande, Mato Grosso do Sul, Brazil; II Universidade Federal do Vale do Jequitinhonha e Mucuri. Diamantina, Minas Gerais, Brazil; III Universidade Federal de Mato Grosso do Sul. Campo Grande, Mato Grosso do Sul, Brazil

**Keywords:** Primary Health Care, Family Development Planning, Contraception, Intrauterine Devices, Effective Access to Health Services., Atención Primaria de Salud, Planificación Familiar, Anticoncepción, Dispositivos Intrauterinos, Acceso Efectivo a los servicios de Salud.

## Abstract

**Objective:**

to assess intrauterine device (IUD) management by nurses in Primary Health Care.

**Method:**

a descriptive and cross-sectional study. Thirty-one nurses qualified to insert IUDs in Mato Grosso do Sul participated. Data were collected online. Frequency analyses and the binomial test were performed using SPSS 24.0, with a 5% significance level.

**Results:**

the majority (83.9%) were female, with a mean age of 37.3 years (±1.35). Of the total, 87.1% had an equipped nursing office and 93.5% used care protocols in their procedures. There was significance in the variables “requests imaging tests”, “prescribes medications” and “performs educational activities” (p<0.001). The monthly mean of insertions was 20.68 (±4.82).

**Conclusion:**

IUD management can be improved with investments in physical and material structures. Nurses are also human resources capable of contributing to the reduction of unplanned pregnancies and their consequences.

## RESUMEN

**Objetivo:**

evaluar el manejo del dispositivo intrauterino (DIU) por parte de enfermeros en la Atención Primaria de Salud.

**Método:**

estudio descriptivo y transversal. Participaron 31 enfermeros capacitados para insertar DIU en Mato Grosso do Sul. Los datos fueron recolectados en línea. Los análisis de frecuencia y la prueba binomial se realizaron mediante SPSS 24.0, con un 5% de significancia.

**Resultados:**

la mayoría (83,9%) son mujeres, con edad promedio de 37,3 años (±1,35). Del total, el 87,1% cuenta con consultorio de enfermería equipado y el 93,5% utiliza protocolos de atención en su conducta. Hubo significancia en las variables “pide pruebas de imagen”, “prescribe medicamentos” y “realiza actividades educativas” (p<0,001). El promedio mensual de inserciones fue de 20,68 (±4,82).

**Conclusión:**

el manejo del DIU se puede mejorar con inversiones en estructuras físicas y materiales. Los enfermeros también son recurso humano capaz de contribuir a la reducción de los embarazos no planificados y sus consecuencias.

## INTRODUCTION

In Brazil, less than 2% of women of childbearing age use a long-term contraceptive method^(1)^, whether it be an intrauterine device (IUD) or a hormonal contraceptive implant. This can have consequences such as unplanned pregnancies due to failures in the use of contraceptives that depend on the woman’s actions, induced abortions and maternal deaths related to pregnancy termination.

Research carried out by the *Fundação Oswaldo Cruz* (Fiocruz), with the participation of 24 thousand women, showed that 55.4% of women who became pregnant were not planning to have children, and this national percentage is above the world mean of 40% of unplanned pregnancies^(2)^.

According to the Pan American Health Organization (PAHO), approximately 303,000 women die each year from complications related to pregnancy, childbirth and/or the postpartum period^(3)^. There are socioeconomic inequities among the determining factors for maternal mortality, and nine out of ten deaths would be avoidable if measures and recommendations from bodies such as the World Health Organization were implemented, such as universal access to contraceptive methods.

There are potential barriers to access to family/reproductive planning services, which hinder women’s process of achieving sexual and reproductive rights. A study identified organizational barriers related to the absence or non-use of IUD insertion protocols, such as excessive insertion criteria that are often unnecessary, limitations on the role of nurses, prior scheduling for the procedure, adoption of certain clinical conditions of women that may make IUD insertion impossible, and conditions not supported by scientific evidence^(4)^. This is a situation that has prevented accessible family/reproductive planning free from the impact of unintended pregnancy.

It is worth noting that Brazil is one of the countries that has made a commitment to the United Nations (UN) through the Sustainable Development Goals (SDGs). Thus, the country has the goal of reducing the maternal mortality rate to less than 30 deaths per 100,000 live births by 2030, in addition to ensuring universal access to sexual and reproductive healthcare services^(5)^. Therefore, it is essential to develop effective actions and strategies to improve health indicators at a national level.

In this context, Law 14,443 of September 2, 2022^(6)^, promoted changes to Law 9,263/1996, which made the criteria for performing tubal ligation and vasectomy more accessible. However, such legislation focused on definitive methods of birth control, but did not encourage the adoption of long-acting and easily reversible contraceptive methods, such as IUD.

In the context of legislation, Brazil is a model for other countries, since family/reproductive planning is a right established in the Federal Constitution, and is guaranteed by specific laws and regulations by the Ministry of Health, among other public institutions. However, the majority of pregnancies are still unplanned, which requires studies to identify the need to improve the quality of reproductive healthcare^(7)^.

Brazilian nurses’ contributions in expanding access to contraceptive methods for women and men, especially long-term contraceptives, stand out. Resolution 690/2022 of the Federal Nursing Council^(8)^ regulated nurses to provide information regarding family/reproductive planning policy and apply eligibility criteria for a person’s decision regarding the best method to use. IUD management, procedures involving health education, insertion, review, removal, among others are considered.

By increasing the number of nurses qualified to handle IUDs, women’s access to and adherence to long-term contraceptive methods will increase, thus favoring birth control. By reducing unplanned pregnancies, it is possible to reduce maternal mortality and improve the living conditions of women and their families.

Taking into account the relevance of the topic and, in order to contribute to the expansion of qualified human resources for contraceptive method management, the objective was to assess IUD management by nurses in Primary Health Care (PHC).

## OBJECTIVE

To assess IUD management by nurses in PHC.

## METHODS

### Ethical aspects

The study was conducted in accordance with national and international ethics guidelines. It was approved by the Research Ethics Committee of Fiocruz Brasília.

### Study design, period and place

This is a descriptive, cross-sectional study with quantitative analysis. Data collection was carried out from July to September 2023 in 12 municipalities of Mato Grosso do Sul: Antônio João, Aral Moreira, Campo Grande, Chapadão do Sul, Deodápolis, Dourados, Iguatemi, Itaquiraí, Nova Andradina, Paranhos, Sete Quedas and Tacuru.

### Population or sample: inclusion and exclusion criteria

A total of 34 nurses qualified to insert copper IUDs were identified in 12 of the 79 municipalities, or 15% of the cities in the state of Mato Grosso do Sul. However, as three refused to participate, the study had 31 participants. From this point on, the expression “copper” will not be mentioned, to avoid repetition. All participants worked in the public healthcare service, predominantly in the Family Health Strategy.

Non-probabilistic and convenience sampling was adopted in the selection of participants. All nurses qualified by the Regional Nursing Council (In Portuguese, *Conselho Regional de Enfermagem* - Coren) to manage IUDs between 2020 and 2023 were included. In turn, those who did not respond to the data collection form after three attempts were excluded.

### Study protocol

The Informed Consent Form was obtained from all professionals involved in the study through an online application. The procedures for data collection involved: a) requesting authorization for the study from Coren; b) creating a WhatsApp group with nurses qualified to manage IUDs to invite them to participate voluntarily; c) sending an online form developed by the researchers on Google Forms for individual responses, at the time, expressing agreement to the Free and Informed Consent Form, with a mean response time of 15 minutes; d) contact by cell phone to clarify any doubts in the answers; e) searching the *Sistema de Informação em Saúde Para a Atenção Básica* (SISAB) to identify the number of records of IUD insertions in PHC from 2020 to 2023.

The instrument used for data collection combined variables in the style of Donabedian’s assessment triad^(9)^, namely structure, process and outcomes. For structure analysis, variables related to women’s access to the method were used as well as the flow of care and supply. Finally, they obtained the variables related to the outcome through information regarding the number of insertions, expulsions, reinsertions and removals of the IUD. Two open-ended questions were included: what strategies contribute to women’s adherence to IUD use? What factors make it difficult to increase the number of women who adhere to this type of long-acting reversible contraception (LARC) method?

### Analysis of results, and statistics

The data were entered into Excel spreadsheets, following the double entry technique. They were subsequently analyzed using descriptive and nonparametric statistics. The assessment of the difference between the percentage of positive responses from professionals observed and the expected percentage (100%) was performed using the binomial test, which measures the proportion of success observed in the sample. For this purpose, the Statistical Package for the Social Sciences (SPSS) version 24.0 was used, considering a 5% significance level. The results were described in percentages, means and respective standard deviations (±), illustrated in tables and graphs.

## RESULTS

Most of nurses who participated were female (83.9%), with a mean age of 37.3 years (±1.35) and a mean time since graduation of 12.2 years (±1.1). Of the total, 37.2% had graduate degrees related to women’s health. The mean time working in PHC was 9.3 years (±1.29) and 1.8 years (±0.26) since qualification in IUD management.

Regarding the minimum structure required for IUD insertion, it is observed that not all locations have the same resources, as illustrated in Table 1.

**Table 1:** Analysis of structures for managing the intrauterine device reported by 31 nurses. Mato Grosso do Sul, Brazil, 2023

Variable	n (%)	Mean
Equipped nursing office		
Yes	27 (87.1)	
No	4 (12.9)	
Has materials of constant consumption		
Yes	27 (87.1)	
No	4 (12.9)	
Has X-ray and ultrasound		
Yes	100.0 (31.0)	
Has computed tomography Yes No	5 (16.1) 26 (83.9)	
Mean of kits with tweezers		2.77±0.39^*^

Note:

* Standard error of the mean.

*Source: SISAB.*

Data related to the work process are presented in Table 2 and reveal that, even with the possibility of taking actions based on care protocols, made available by public institutions, such as the Ministry of Health and Coren, not all nurses use such frameworks.

**Table 2:** Analysis of processes in intrauterine device management reported by 31 nurses. Mato Grosso do Sul, Brazil, 2023

Variable	n (%)
The intrauterine device insertion follows the Ministry of Health recommendations	
Yes	18 (58.1)
No	13 (41.9)
Uses a protocol to insert the intrauterine device	
Yes	29 (93.5)
No	2 (6.5)
There is a local protocol for handling incidents	
Yes	22 (71.0)
No	9 (29.0)
Performs copper intrauterine device insertions in the unit where they work	
Yes	21 (67.7)
No	10 (32.3)
Inserts another method besides the intrauterine device	
Yes	2 (6.5)
No	29 (93.5)
In the municipality, there are other professionals trained to insert the intrauterine device	
Yes	31 (100.0)
Scheduling method for nursing consultation	
Scheduled	19 (61.3)
Spontaneous	7 (22.6)
Did not answer	5 (16.1)

Although all participants were qualified to use IUDs, 12.9% stopped working directly in healthcare to take on administrative roles, and 19.3%, although they work in healthcare, do not insert the method, a situation that compromises access to the long-acting method. However, IUD management is not an activity exclusive to nurses, and all locations have other professionals who also assist women. In most cases, access to professionals requires an appointment, given the limited materials available.

Participants responded that 100.0% of municipalities have ultrasound and X-ray tests, but there were divergences regarding nurses’ autonomy to request imaging tests and prescribe medications. It is worth noting that, of the total number of participants, only 41.9% reported prescribing analgesics, 38.7% anti-inflammatories, and 6.5% antibiotics.

The data showed significance (p<0.001), through binomial analysis, in the variables “requests imaging tests”, “prescribes medications” and “performs educational activities”, with the majority of participants (90.3%) reporting requesting a transvaginal ultrasound test to assess the positioning of the IUD and 61.3% prescribing medications, if a woman needs to treat pain. In contrast, 19.4% do not provide guidance or clarify doubts in operational groups.

As for IUD insertion, Table 3 shows that not all women of childbearing age have access to the method.

**Table 3:** Description of groups receiving intrauterine device insertion by 31 nurses. Mato Grosso do Sul, Brazil, 2023

Variable	n (%)
Multiparous	
Yes	31 (100.0)
Nulliparous	
Yes	26 (83.9)
No	5 (16.1)
Adolescents	
Yes	21 (67.7)
No	10 (32.3)

Table 4 presents the number of nurses who claim to have knowledge or lack of knowledge of situations related to the use of IUDs, such as expulsion and requests to stop using them, in addition to variables related to the management stages in assisting women experienced in these professionals’ work.

**Table 4:** Analysis of outcomes regarding the intrauterine device management reported by 31 nurses. Mato Grosso do Sul, Brazil, 2023

Variable	n (%)	Mean
Mean monthly insertions		20.68±4.82^*^
Was the intrauterine device expelled?		
Yes	16 (51.6)	
No	15 (48.4)	
Mean number of expulsions		1.32±0.31^*^
Was the intrauterine device reinserted?		
Yes	12 (38.7)	
No	19 (61.3)	
Mean number of reinsertions		0.58±0.17^*^
Was the intrauterine device discontinued?		
Yes	16 (51.6)	
No	15 (48.4)	
Mean number of women who stopped using		2.32±1.00^*^

Note:

* Standard error of the mean.

In relation to strategies that facilitate IUD insertion, the highest frequency (29.0%) was health education, followed by dissemination by community health agents (12.9%) and the fact that the procedure is also performed by nurses (12.9%). Among the factors that hinder the increase in the number of women using this LARC, from nurses’ perspective, were lack of knowledge among the population (32.3%), myths surrounding the method (25.8%) and the scarcity of materials (12.9%).

**Figure 1:**
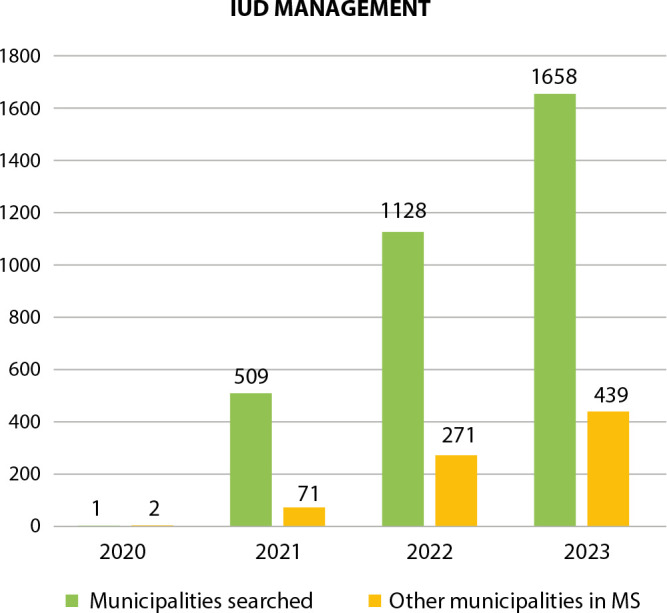
Quantitative analysis of intrauterine device management in Primary Health Care. Mato Grosso do Sul, Brazil, 2020 to 2023

A search was carried out in SISAB, during the stipulated period, using the SIGTAP procedure code (0301040141), which refers to the insertion of an IUD, the data for which are shown in Figure 1.

## DISCUSSION

The predominance of female participants is similar to that reported by PAHO^(10)^, because even with the growing male presence in nursing, it is still a predominantly female profession, a scenario that could favor the expansion of the supply of various contraceptive methods, since most of them are for women’s use, such as IUD.

The fact that nurses are young and have more than ten years of professional experience could benefit the implementation of family planning policy^(6)^, given the potential of these professionals to be multipliers for the qualification of peers, especially in the same workplace, such as the PHC model in Florianópolis, in which most health units offer IUD insertion through the Brazilian Health System (In Portuguese, *Sistema Único de Saúde* - SUS)^(11)^.

The data showed that qualification in the management of one of the types of LARC did not occur within the scope of the specialization focused on women’s health, which constitutes a warning to *lato sensu* graduate courses to offer teaching of professional skills that correspond to the degree obtained and to the needs of society, as they are a level of education higher than undergraduate studies and are intended to specialize professionals in a specific area^(12)^.

The structure axis analysis revealed the need for investments by managers, as they are responsible for providing ideal conditions for safe assistance to society^(13)^. There are locations with limited physical space, and appointments are interspersed with other professionals due to the shortage of offices. In addition, the limited number of clamps available for IUD insertion limits both assistance to women and the implementation of public health policy and nursing practice, in accordance with professional and health laws and standards.

Even with the difficulties faced by healthcare services, studies reveal the commitment of Brazilian nurses to promote women’s access to family/reproductive planning, as in the present study and in Minas Gerais^(14)^, Florianópolis^(11)^ and São Paulo^(15)^, states that also have IUD insertion by nurses^(16)^. Therefore, these locations are examples so that others can implement the same service and, with this, benefit society.

Studies have shown no difference in IUD insertion performed by doctors or nurses^(15,17,18)^, and suggest that healthcare services invest in training nurses to perform IUD insertions, in line with the Ministry of Health guidelines^(1)^.

Investing in training nurses to manage IUDs, in addition to increasing access for women, reduces pent-up demand, considering the mean number of insertions presented in this study. However, organizational barriers^(4)^ make it difficult to increase, more quickly, the number of women who regulate procreation.

In this scenario, access to long-term contraceptive methods such as the IUD is characterized by the existence of professionals qualified to carry out its management, and relies on the assistance of tests to identify any complications, although events that require interventions are rare^(14,15)^. We would like to emphasize that IUD insertion is not conditional on the performance of tests, as recommended by the Ministry of Health^(1)^.

It is important to note that the right to family/reproductive planning is guaranteed to citizens by the Federal Constitution, and has had a specific law since 1996^(19)^. However, municipal and state managers need to create strategies so that women and men can exercise their rights to have or not have children, and also to reduce the total number of induced abortions and the number of deaths of women related to unwanted pregnancies.

It is worth noting that all Brazilian municipalities have the SUS, and there are enough nurses to increase the number of people qualified to handle the IUD, a highly effective and safe method^(20)^. Therefore, the country has human resources capable of overcoming the challenges in meeting the goals established in the third SDG proposed by the UN^(5)^, with emphasis on reducing maternal and infant mortality and universal access to sexual and reproductive healthcare services.

In relation to the work process, most nurses use care protocols both for decision-making and for systematizing care. Based on the premise that standardization of procedures promotes care based on scientific evidence and reduces the possibility of complications, when procedures are performed randomly, they put the safety of patients and professionals at risk, i.e., they favor damage to health and generate professional dilemmas^(21)^.

The study identified negative aspects in variables considered essential for qualified IUD management. The situation found shows that some nurses fail to exercise professional autonomy by not requesting imaging tests, such as ultrasound, or prescribing drug treatment, such as analgesics, when necessary, and end up offering fragmented care, which may be related to gaps in their training. The authors emphasize the importance of continuing education and matrix support, with the aim of improving both professional qualification and problem-solving capacity in PHC^(22)^.

It is true that the insertion of an IUD is not conditional on the availability of tests, however it is desirable to have resources to identify possible complications, such as an IUD inserted outside the uterine cavity, among others, and within the scope of nursing consultation, Law 7.498/86 legitimizes nursing actions^(23)^. Therefore, the request for tests and the prescription of medications are reproductive healthcare actions performed by nurses^(23)^.

There are places that have care protocols. When identifying unexpected events, such as metrorrhagia, intense pain, fever, etc., nurses refer the woman for medical assessment and treatment. Therefore, it is essential that women are informed about the risks, benefits, advantages, disadvantages and effectiveness of the most diverse contraceptive methods^(24)^.

A law that deals with family planning policy^(6,19)^ assigns to the State the duty to promote preventive and educational actions through information, methods and techniques of birth control. Therefore, it is up to professionals working in the SUS to provide sexual and reproductive education on a daily basis, not only to comply with a legal obligation, but as a strategy to publicize the resources available for contraception, identify the need to demystify popular knowledge surrounding fertility control, and welcome women, men and/or couples, from the perspective of global and comprehensive healthcare^(25)^.

Regarding access to IUDs by adolescents and nulliparous women, the context is challenging, due to the fact that there are professionals who do not assist these groups and who are in disagreement with public policies, such as comprehensive care for adolescent health, comprehensive care for women’s health and the law itself that deals with family planning^(6,19)^. It is necessary to identify the reasons for this segregating reality in order to correct this situation.

In this regard, it is up to Coren to provide the necessary clarifications and guidance to nursing professionals, based on the legislation that governs the profession, so that supervision is educational, supportive and preventative of punishments for negligent conduct^(23)^.

The results made clear nurses’ daily work, marked by the transformation in women’s lives, since the copper IUD can be used for up to ten years^(26)^ and promotes full sexual health and the realization of personal projects, such as acquiring a profession or material goods that can improve quality of life, benefiting from pregnancy planning.

Women were assured of comprehensive care in sexual and reproductive health assistance, as evidenced by the activities carried out, which were not restricted to just inserting the IUD, but also providing care in situations of expulsion of the device and its removal, when requested.

In relation to IUD expulsion, this is a clinical situation that can occur within the first year of use, with common rates of around 10% of insertions being observed^(24)^. A study carried out with 75 women who received a copper IUD in the period 30 to 45 days postpartum showed that the expulsion rate was around 1.3%, increasing to 5.3% over the first 12 months of use^(25)^. In the United States of America, the successful insertion rate for nurses and other healthcare professionals was approximately 4.8 times higher after the training program^(27)^.

In this study, the expulsion rate was 2%, demonstrating a positive outlook on the adaptation of the method. These data coincide with international findings in Australia, where, of 207 insertions supervised by the training professional, 91% of insertions were successful and did not have direct supervision^(28)^.

It is noteworthy that locations that implement or expand family/reproductive planning services will be contributing to female empowerment and access to the service, which helps reduce maternal mortality^(5,11,29)^.

Participants shared experiences used to raise awareness among women about IUD use, such as community health workers, in order to highlight multidisciplinary work. In this sense, it is important to promote the training of all workers involved to improve the production of health work^(29)^.

The data recorded in SISAB covered reproductive healthcare, and it is noteworthy that there were a total of women who received IUDs in the 12 municipalities where the participants work, in contrast to the other 67 cities in the state of Mato Grosso do Sul. This information makes us wonder whether all professionals keep proper records of care, an action of great importance for measuring indicators of target 3.7 of the SDGs^(5)^.

The results are in addition to other studies^(11,14,28)^, which demonstrated the expansion of access to IUDs through an increase in the sexual and reproductive health workforce and the potential to reduce maternal mortality related to unplanned and unwanted pregnancies.

### Study limitations

As limitations, the convenience sample was considered, in which nurses who were qualified by Coren in the 2018 to 2023 management participated, not being representative of the entire state of Mato Grosso do Sul. However, the results obtained are similar to those of other national studies^(11,14,28)^, which also showed the expansion of access to IUDs by PHC nurses, indicating possibilities that can be reproduced in other locations for investment in the qualification of nurses in LARC management.

### Contributions to nursing, health or public policies

Training nurses to manage IUDs represents human resources available for actions that contribute to the reduction of unwanted pregnancies, abortions and maternal mortality. It fostered the national commitment to the UN to ensure universal access to sexual and reproductive healthcare services and supplies by 2030, including reproductive planning, access to information and education, as well as the integration of reproductive health into national strategies and programs.

## CONCLUSION

Training nurses to manage long-term contraceptive methods is a strategic action to expand the workforce in favor of sexual and reproductive health, especially through the SUS. Furthermore, the study is similar to other national and international studies, with similar rates, demonstrating competence for the appropriate IUD management.

It is up to managers to implement public health policies to provide the necessary structures for a safe, effective work process that is in line with scientific and legal evidence.

This study represents an innovation in Brazilian nursing practices, as it revealed that it is possible to improve reproductive health and, therefore, reduce unplanned pregnancies and their consequences.
