# Supplementary material for: Intrauterine device management by nurses in Primary Health Care
Source: Rev Bras Enferm. 2025 Jun 13;78(Suppl 1):e20240340. doi: 10.1590/0034-7167-2024-0340 (PMC12165715; doi:10.1590/0034-7167-2024-0340)
Supplement: Translation

# Translation: Manejo do dispositivo intrauterino por enfermeiros na Atenção Primária à Saúde

**DOI:** 10.1590/0034-7167-2024-0340pt

**Authors:** Eliane Kelly Ribeiro da Silva, Helisamara Mota Guedes, Débora Dupas Gonçalves do Nascimento, Albert Schiaveto de Souza, Sebastião Junior Henrique Duarte

**Affiliations:** I Fundação Oswaldo Cruz. Campo Grande, Mato Grosso do Sul, Brasil; II Universidade Federal do Vale do Jequitinhonha e Mucuri. Diamantina, Minas Gerais, Brasil; III Universidade Federal de Mato Grosso do Sul. Campo Grande, Mato Grosso do Sul, Brasil

**Keywords:** Atenção Primária à, Saúde, Planejamento Familiar, Anticoncepção, Dispositivos Intrauterinos, Acesso Efetivo aos Serviços de Saúde.

## Abstract

**Objetivo:**

avaliar o manejo do dispositivo intrauterino (DIU) por enfermeiros na Atenção Primária à Saúde.

**Método:**

estudo descritivo e transversal. Participaram 31 enfermeiros habilitados para a inserção do DIU no Mato Grosso do Sul. Dados foram coletados *online*. Realizaram-se as análises das frequências e teste binomial pelo SPSS 24.0, com 5% de significância.

**Resultados:**

a maioria (83,9%) é do sexo feminino, com média de 37,3 anos (±1,35). Do total, 87,1% dispõem de consultório de enfermagem equipado e 93,5% utilizam protocolos assistenciais em suas condutas. Houve significância nas variáveis “solicita exames de imagem”, “prescreve medicamentos” e “realiza atividades educativas” (p<0,001). A média mensal de inserções foi de 20,68 (±4,82).

**Conclusão:**

o manejo do DIU pode ser melhorado com investimentos em estruturas físicas e materiais. Enfermeiros também são recursos humanos capazes de contribuir com a redução de gravidez não planejada e suas consequências.

## INTRODUÇÃO

No Brasil, menos de 2% das mulheres em idade fértil faz uso de método contraceptivo de longa duração^(1)^, seja o dispositivo intrauterino (DIU) ou o implante hormonal anticoncepcional. Com isso, é possível que haja consequências, como a gravidez não planejada por falhas no uso de contraceptivos que dependem da ação da mulher, abortos provocados e morte materna relacionada à interrupção da gestação.

Pesquisa realizada pela Fundação Oswaldo Cruz (Fiocruz), com a participação de 24 mil mulheres, apontou que 55,4% das mulheres que engravidaram não estavam planejando ter filhos, e este percentual nacional está acima da média mundial, de 40% de gestações não planejadas^(2)^.

De acordo com a Organização Pan-Americana da Saúde (OPAS), anualmente morrem aproximadamente 303 mil mulheres por complicações relacionadas à gravidez, ao parto e/ou puerpério^(3)^. Existem iniquidades socioeconômicas entre os fatores determinantes para a mortalidade materna, e nove a cada dez mortes seriam evitáveis se medidas e recomendações de órgãos como a Organização Mundial da Saúde fossem implementadas, como o acesso universal a métodos contraceptivos.

Verifica-se a possibilidade de barreiras no acesso aos serviços de planejamento familiar/reprodutor, o que fere o processo de conquista das mulheres em relação aos direitos sexuais e reprodutivos. Estudo identificou barreiras organizacionais relativas à ausência ou não utilização de protocolos de inserção do DIU, como excesso nos critérios de inserção, sendo, muitas vezes, desnecessários, limitação da atuação do enfermeiro, agendamento prévio para realização do procedimento, adoção de determinadas condições clínicas da mulher que podem impossibilitar a inserção do DIU e condições sem respaldo em evidências científicas^(4)^. Trata-se de situação que tem impedido o planejamento familiar/reprodutor acessível livre de impacto em torno da gestação não intencional.

Ressalta-se que o Brasil é um dos países que assumiu compromisso junto à Organização das Nações Unidas (ONU) por meio dos Objetivos de Desenvolvimento Sustentável (ODS). Assim, o país tem a meta de até o ano de 2030 reduzir a taxa de mortalidade materna para menos de 30 mortes por 100.000 nascidos vivos, além de assegurar o acesso universal aos serviços de saúde sexual e reprodutiva^(5)^. Portanto, torna-se fundamental o desenvolvimento de ações e estratégias eficazes para melhorar os indicadores de saúde em âmbito nacional.

Nesse contexto, a Lei n° 14.443, de 2 de setembro de 2022^(6)^, promoveu alterações na Lei n° 9.263/1996, que tornou mais acessíveis os critérios para a realização da laqueadura e da vasectomia. Contudo, tais legislações centraram-se nos métodos definitivos de controle da natalidade, mas não estimularam a adoção dos métodos contraceptivos de longa duração e fácil reversão, como o DIU.

No contexto da legislação, o Brasil é modelo para outros países, visto o planejamento familiar/reprodutor ser direito estabelecido na Constituição Federal, estar assegurado em leis específicas e regulamentações pelo Ministério da Saúde, entre outras instituições públicas. Contudo, a maioria das gestações ainda não é planejada, o que enseja a realização de estudos que identifiquem as necessidades de melhorar a qualidade de atenção à saúde reprodutiva^(7)^.

Destacam-se as contribuições de enfermeiros brasileiros na ampliação do acesso às mulheres e homens aos métodos contraceptivos, em especial aos de longa duração. A Resolução n° 690/2022 do Conselho Federal de Enfermagem^(8)^ regulamentou os enfermeiros a prestar informações a respeito da política de planejamento familiar/reprodutor e aplicar os critérios de elegibilidade para a decisão da pessoa quanto ao melhor método a usar. Consideram-se o manejo do DIU, os procedimentos que envolvem a educação em saúde, inserção, revisão, remoção, entre outros.

Ao se ampliar o número de enfermeiros habilitados para o manejo do DIU, aumentam-se o acesso e a adesão de mulheres a métodos contraceptivos de longa duração, favorecendo o controle da natalidade. Com a redução de gravidezes não planejadas, é possível reduzir a mortalidade materna e melhorar as condições de vida das mulheres e seus familiares.

Tomando a relevância da temática e, no sentido de contribuir com a ampliação de recursos humanos qualificados para o manejo dos métodos contraceptivos, teve-se como objetivo avaliar o manejo do DIU por enfermeiros na Atenção Primária à Saúde (APS).

## OBJETIVO

Avaliar o manejo do DIU por enfermeiros na APS.

## MÉTODOS

### Aspectos éticos

O estudo foi conduzido de acordo com as diretrizes de ética nacionais e internacionais. Recebeu aprovação pelo Comitê de Ética em Pesquisa da Fiocruz Brasília.

### Desenho, período e local do estudo

Trata se de estudo descritivo, transversal, de análise quantitativa. A coleta de dados foi realizada no período de julho a setembro de 2023 em 12 municípios de Mato Grosso do Sul: Antônio João, Aral Moreira, Campo Grande, Chapadão do Sul, Deodápolis, Dourados, Iguatemi, Itaquiraí, Nova Andradina, Paranhos, Sete Quedas e Tacuru.

### População ou amostra: critérios de inclusão e exclusão

Identificou-se o total de 34 enfermeiros, habilitados para a inserção do DIU de cobre, em 12 dos 79 municípios, ou seja, 15% das cidades do estado de Mato Grosso do Sul. No entanto, como três se recusaram a participar, o estudo contou com 31 participantes. A partir daqui, não será mencionada a expressão “de cobre”, para evitar repetições. Todos os participantes atuavam no serviço público de saúde, predominantemente na Estratégia Saúde da Família.

Na seleção dos participantes, adotou-se a amostragem não probabilística e por conveniência. Incluíram-se todos os enfermeiros habilitados pelo Conselho Regional de Enfermagem (Coren) para o manejo do DIU entre os anos de 2020 e 2023. Por sua vez, excluíram-se os que não responderam ao formulário para a coleta de dados, após três tentativas.

### Protocolo do estudo

O Termo de Consentimento Livre e Esclarecido foi obtido de todos os profissionais envolvidos no estudo através de aplicativo *online*. Os procedimentos para a coleta de dados envolveram: a) solicitação de autorização do estudo ao Coren; b) criação de um grupo de *WhatsApp* com os enfermeiros habilitados para o manejo do DIU, para convidá-los a participar voluntariamente; c) envio de formulário *online* desenvolvido pelos pesquisadores no *Google Forms* para resposta individual, na oportunidade, a manifestação de concordância no Termo de Consentimento Livre e Esclarecido, com o tempo médio de resposta de 15 minutos; d) contato por celular para esclarecimento de eventuais dúvidas nas respostas; e) busca no Sistema de Informação em Saúde Para a Atenção Básica (SISAB) para identificar o número de registros de inserções do DIU na APS no período de 2020 a 2023.

O instrumento utilizado na coleta de dados combinou variáveis no estilo da tríade de avaliação de Donabedian^(9)^, sendo elas estrutura, processo e resultado. Para a análise da estrutura, utilizaram-se variáveis relacionadas ao acesso da mulher ao método, assim como fluxo de atendimento e oferta. Por fim, obtiveram as variáveis relacionadas ao resultado por meio de informações referentes ao quantitativo de inserções, expulsões, reinserções e remoção do DIU. Incluíram-se duas questões abertas: quais as estratégias que contribuem à adesão de mulheres ao uso do DIU? Quais os fatores que dificultam ampliar o número de mulheres que aderem a esse tipo de método de contracepção reversível de longa duração (cuja sigla em inglês é LARC)?

### Análise dos resultados e estatística

Os dados foram digitados em planilhas do programa *Excel*, seguindo a técnica de dupla entrada. Posteriormente, receberam análise por meio da estatística descritiva e não paramétrica. A avaliação da diferença entre o percentual de respostas positivas dos profissionais observados e aquele esperado (100%) foi realizada por meio do teste binomial, que mede a proporção de sucesso observadas na amostra. Para tanto, contou-se com o auxílio do *software Statistical Package for the Social Sciences* (SPSS), versão 24.0, considerando 5% de significância. Os resultados foram descritos em percentuais, médias e respectivos desvios padrões (±), ilustrados em tabelas e gráfico.

## RESULTADOS

A maior parte dos enfermeiros que participaram era do sexo feminino (83,9%), com média de idade de 37,3 anos (±1,35) e tempo de graduação de 12,2 anos (±1,1). Do total, 37,2% possuem pós-graduação relacionada à saúde da mulher. A média do tempo de trabalho na APS foi de 9,3 anos (±1,29) e 1,8 anos (±0,26) da habilitação em manejo do DIU.

Com relação à estrutura minimamente necessária para a inserção do DIU, observa-se que nem todas as localidades possuem os mesmos recursos, conforme ilustrado na Tabela 1.

**Tabela 1:** Análise das estruturas para o manejo do dispositivo intrauterino referidas por 31 enfermeiros. Mato Grosso do Sul, Brasil, 2023

Variável	n (%)	Média
Consultório de enfermagem equipado		
Sim	27 (87,1)	
Não	4 (12,9)	
Possui materiais de consumo constante		
Sim	27 (87,1)	
Não	4 (12,9)	
Dispõe de radiografia e ultrassom		
Sim	100,0 (31,0)	
Dispõe de tomografia computadorizada Sim Não	5 (16,1) 26 (83,9)	
Média de *kits* com pinças		2,77±0,39^*^

Nota:

* Erro padrão da média.

*Fonte: SISAB.*

Dados relacionados ao processo de trabalho são apresentados na Tabela 2 e revelam que, mesmo com a possibilidade de tomar condutas baseadas em protocolos assistenciais, disponibilizados por instituições públicas, como o Ministério da Saúde e o Coren, nem todos os enfermeiros utilizam tais referenciais.

**Tabela 2:** Análise dos processos no manejo do dispositivo intrauterino referidos por 31 enfermeiros. Mato Grosso do Sul, Brasil, 2023

Variável	n (%)
A inserção do dispositivo intrauterino segue as recomendações do Ministério da Saúde	
Sim	18 (58,1)
Não	13 (41,9)
Utiliza algum protocolo para a inserção do dispositivo intrauterino	
Sim	29 (93,5)
Não	2 (6,5)
Existe protocolo local para manejo de intercorrências	
Sim	22 (71,0)
Não	9 (29,0)
Realiza as inserções de dispositivo intrauterino de cobre na unidade onde trabalha	
Sim	21 (67,7)
Não	10 (32,3)
Insere outro método além de dispositivo intrauterino	
Sim	2 (6,5)
Não	29 (93,5)
No município, há outros profissionais capacitados para inserção do dispositivo intrauterino	
Sim	31 (100,0)
Modo de agendamento para a consulta de enfermagem	
Programada	19 (61,3)
Espontânea	7 (22,6)
Não respondeu	5 (16,1)

Ainda que todos os participantes foram habilitados ao manejo do DIU, 12,9% deixaram de trabalhar diretamente na assistência para assumir funções administrativas, e 19,3%, embora estejam na assistência, não inserem o método, situação que compromete o acesso ao método de longa duração. Contudo, o manejo do DIU não é atividade exclusiva de enfermeiros, e todas as localidades contam com outros profissionais que também assistem as mulheres. Na maioria das vezes, o acesso aos profissionais requer agendamento, tendo em vista a limitação de materiais disponíveis.

Os participantes responderam que 100,0% dos municípios contam com os exames de ultrassonografia e raio-X, porém houve divergências quanto à autonomia de enfermeiros solicitarem exames de imagens e prescreverem medicamentos. Cabe destacar que, do total de participantes, apenas 41,9% referiram prescrever analgésicos, 38,7%, anti-inflamatórios, e 6,5%, antibióticos.

Os dados apresentaram significância (p<0,001), por meio da análise binomial, nas variáveis “solicita exames de imagem”, “prescreve medicamentos” e “realiza atividades educativas”, sendo que a maioria dos participantes (90,3%) referiu solicitar o exame de ultrassonografia transvaginal para a avaliação do posicionamento do DIU e 61,3% prescrevem medicamentos, caso a mulher tenha necessidade de tratar a dor. Em contraposição, 19,4% não promovem orientações e esclarecimento de dúvidas em grupos operativos.

Em relação à inserção do DIU, a Tabela 3 mostra que nem toda mulher em idade fértil tem acesso ao método.

**Tabela 3:** Descrição dos grupos que recebem a inserção do dispositivo intrauterino por 31 enfermeiros. Mato Grosso do Sul, Brasil, 2023

Variável	n (%)
Multíparas	
Sim	31 (100,0)
Nulíparas	
Sim	26 (83,9)
Não	5 (16,1)
Adolescentes	
Sim	21 (67,7)
Não	10 (32,3)

Na Tabela 4, é apresentado o quantitativo de enfermeiros que afirmam ter o conhecimento ou desconhecimento de situações relacionadas ao uso do DIU, como a expulsão e a solicitação de interrupção do uso, além de variáveis relacionadas às etapas do manejo na assistência às mulheres vivenciadas no trabalho desses profissionais.

**Tabela 4:** Análise dos resultados referentes ao manejo do dispositivo intrauterino referidos por 31 enfermeiros. Mato Grosso do Sul, Brasil, 2023

Variável	n (%)	Média
Média mensal de inserções		20,68±4,82^*^
Houve expulsão do dispositivo intrauterino?		
Sim	16 (51,6)	
Não	15 (48,4)	
Média de expulsões		1,32±0,31^*^
Houve reinserção de dispositivo intrauterino?		
Sim	12 (38,7)	
Não	19 (61,3)	
Média de reinserções		0,58±0,17^*^
Solicitada a interrupção do uso do dispositivo intrauterino?		
Sim	16 (51,6)	
Não	15 (48,4)	
Média de mulheres que interromperam o uso		2,32±1,00^*^

Nota:

* Erro padrão da média.

Quanto às estratégias que facilitam a inserção do DIU, a maior frequência (29,0%) se deu pela educação em saúde, seguida da divulgação pelos agentes comunitários de saúde (12,9%) e pelo fato de o procedimento também ser realizado por enfermeiros (12,9%). Entre os fatores que dificultam a ampliação no número de mulheres para o uso desse LARC, sob a ótica dos enfermeiros, foram citados o desconhecimento por parte da população (32,3%), mitos em torno do método (25,8%) e a escassez de materiais (12,9%).

Realizou-se busca no SISAB, no período estipulado, através do código do procedimento SIGTAP (0301040141), que é referente à inserção de DIU, cujos dados são expressos na Figura 1.

**Figura 1:** Análise quantitativa do manejo do dispositivo intrauterino na Atenção Primária à Saúde. Mato Grosso do Sul, Brasil, 2020 a 2023

## DISCUSSÃO

A predominância de participantes do sexo feminino é semelhante ao divulgado pela OPAS^(10)^, pois, mesmo com a crescente presença masculina na enfermagem, ainda assim configura-se como profissão predominantemente feminina, cenário que pode favorecer a ampliação da oferta dos diversos métodos contraceptivos, pois a maioria deles é para o uso de mulheres, como o DIU.

O fato de as enfermeiras serem jovens e com mais de dez anos de experiência profissional pode beneficiar a implementação da política de planejamento familiar^(6)^, haja vista o potencial dessas profissionais de serem multiplicadoras para a habilitação de pares, principalmente na mesma localidade de trabalho, como o modelo de APS de Florianópolis, em que a maioria das unidades de saúde oferece a inserção do DIU pelo Sistema Único de Saúde (SUS)^(11)^.

Os dados mostraram que a habilitação em manejo de um dos tipos de LARC não ocorreu no âmbito da especialização voltada à saúde da mulher, o que constitui um alerta aos cursos de pós-graduação *lato sensu* para que ofereçam o ensino de competências profissionais que corresponda ao título obtido e às necessidades da sociedade, pois são um nível de ensino superior à graduação e têm a finalidade de especializar o profissional em determinada área^(12)^.

A análise do eixo estrutura revelou a necessidade de investimentos por parte dos gestores, por serem os responsáveis em prover as condições ideais à assistência segura à sociedade^(13)^. Há localidades com limitações de espaço físico, e os atendimentos são intercalados com outros profissionais, devido à escassez de consultórios. Além disso, o reduzido número de pinças disponíveis para a realização da inserção do DIU limita tanto a assistência às mulheres quanto a efetivação da política pública de saúde e o exercício da profissão, em conformidade com as legislações e normas profissionais e sanitárias.

Mesmo com as dificuldades enfrentadas pelos serviços de saúde, estudos revelam o empenho de enfermeiros brasileiros para promover o acesso às mulheres ao planejamento familiar/reprodutivo, a exemplo do presente estudo e de Minas Gerais^(14)^, Florianópolis^(11)^ e São Paulo^(15)^, estados que contam com a inserção do DIU também por enfermeiros^(16)^. Portanto, tais localidades são exemplos para que outras possam implementar o mesmo serviço e, com isso, beneficiar a sociedade.

Estudos mostraram não possuir diferença na inserção do DIU realizada por médicos ou enfermeiros^(15,17,18)^, e sugerem que os serviços de saúde invistam na formação de enfermeiros para realizar inserções de DIU, indo de encontro à orientação do Ministério da Saúde^(1)^.

O investimento na habilitação de enfermeiros para o manejo do DIU, além de ampliar o acesso às mulheres, reduz demanda reprimida, considerando a média de inserções apresentadas neste estudo. No entanto, barreiras organizacionais^(4)^ dificultam aumentar, com mais celeridade, o número de mulheres que regulam a procriação.

Diante desse cenário, o acesso a método contraceptivo de longa duração como o DIU é caracterizado pela existência de profissionais habilitados para realizar seu manejo, e conta com o auxílio de exames para a identificação de alguma intercorrência, embora sejam raros os eventos que requerem intervenções^(14,15)^. Reforçamos que a inserção do DIU não está condicionada à realização de exames, como recomenda o Ministério da Saúde^(1)^.

Ressalta-se que o direito ao planejamento familiar/reprodutor é assegurado pela Constituição Federal aos cidadãos, e possui lei específica desde 1996^(19)^. No entanto, é preciso que os gestores municipais e estaduais criem estratégias para que mulheres e homens possam exercer seus direitos de ter ou não filhos e que, ainda, haja redução no total de abortos provocados e na morte de mulheres relacionada à gestação indesejada.

Cabe destacar que todos os municípios brasileiros contam com o SUS, e existem enfermeiros em número suficiente para ampliar os habilitados a manejar o DIU, um método altamente eficaz e seguro^(20)^. Portanto, o país tem recursos humanos capazes de superar os desafios no cumprimento das metas estabelecidas no terceiro ODS proposto pela ONU^(5)^, com destaque à redução da mortalidade materna e infantil e ao acesso universal aos serviços de saúde sexual e reprodutiva.

Em relação ao processo de trabalho, a maioria dos enfermeiros utiliza protocolos assistenciais tanto para a tomada de decisão quanto para a sistematização da assistência. Partindo-se da premissa de que a padronização de procedimentos promove o cuidado baseado em evidências científicas e reduz as possibilidades de intercorrências, quando as condutas ocorrem de modo aleatório, colocam em risco a segurança do paciente e do profissional, ou seja, favorecem danos à saúde e geram dilema profissional^(21)^.

O estudo identificou aspectos negativos em variáveis consideradas essenciais no manejo qualificado do DIU. A situação encontrada evidencia que alguns enfermeiros deixam de exercer a autonomia profissional ao não solicitarem exames de imagem, como a ultrassonografia, ou prescreverem tratamento medicamentoso, como analgésicos, quando necessário, passando a oferecer uma assistência fragmentada, o que pode estar relacionado a lacunas em sua formação. Os autores reforçam a importância da educação permanente e do matriciamento, na intenção de melhorar tanto a qualificação profissional quanto a resolutividade na APS^(22)^.

É certo que a inserção do DIU não está condicionada à disponibilização de exames, no entanto é desejável contar com recursos para identificação de eventuais intercorrências, a exemplo do DIU inserido fora da cavidade uterina, entre outras, e no âmbito da consulta de enfermagem, a Lei n^o^ 7.498/86 legitima as ações de enfermagem^(23)^. Logo, a solicitação de exames e a prescrição de medicamentos são ações de atendimento à saúde reprodutiva realizadas por enfermeiros^(23)^.

Há localidades que contam com protocolos assistenciais. Ao identificar eventos inesperados, como metrorragia, dor intensa, febre, *etc*., os enfermeiros encaminham a mulher para a avaliação e conduta médica. Por isso, é fundamental que as mulheres sejam esclarecidas com informações a respeito dos riscos, benefícios, vantagens, desvantagens e eficácia dos mais diversos métodos contraceptivos^(24)^.

A lei que trata da política de planejamento familiar^(6,19)^ atribui ao Estado o dever de promover ações preventivas e educativas através de informações, métodos e técnicas de controle da natalidade. Portanto, cabe aos profissionais que atuam no SUS realizar a educação sexual e reprodutiva no cotidiano, não só para cumprir obrigação legal, mas por ser estratégia para divulgar os recursos disponibilizados para a contracepção, identificar a necessidade de desmitificar o saber popular em torno do controle da fecundidade, e acolher a mulher, o homem e/ou o casal, na perspectiva de um atendimento global e integral à saúde^(25)^.

No que se refere ao acesso de adolescentes e das nulíparas ao DIU, o contexto é desafiador, devido a ter profissionais que não assistem esses grupos e que estão em desacordo com as políticas públicas, como a atenção integral à saúde do adolescente, atenção integral à saúde da mulher e da própria lei que trata do planejamento familiar^(6,19)^. É preciso identificar os motivos dessa realidade segregadora, de modo a corrigir essa situação.

Nesse sentido, cabe ao Coren promover os devidos esclarecimentos e a orientação aos profissionais de enfermagem, a partir das legislações que disciplinam a profissão, de modo que a fiscalização seja educativa, solidária e preventiva das punições por condutas negligentes^(23)^.

Os resultados deixaram claro o cotidiano do trabalho dos enfermeiros, marcado pela transformação na vida das mulheres, visto que o DIU de cobre pode ser usado por até dez anos^(26)^ e favorece a saúde sexual plena e a realização de projetos pessoais, como adquirir uma profissão ou bens materiais que possam melhorar a qualidade de vida, usufruindo do planejamento da gravidez.

Às mulheres, foi assegurado o atendimento integral na assistência à saúde sexual e reprodutiva, evidenciado nas atividades realizadas, que não se restringiram somente a introduzir o DIU, mas a prestar atendimento nas situações de expulsão do dispositivo e na sua retirada, quando solicitado.

No que tange à expulsão do DIU, é uma situação clínica que pode ocorrer dentro do primeiro ano de uso, sendo observadas taxas comuns em torno de 10% das inserções^(24)^. Estudo realizado com 75 mulheres que receberam DIU de cobre no período de 30 a 45 dias pós-parto mostrou que a taxa de expulsão girou em torno de 1,3%, aumentando para 5,3% ao longo dos primeiros 12 meses de uso^(25)^. Nos Estados Unidos da América, a taxa de inserção bem-sucedida para enfermeiros e outros profissionais de saúde foi aproximadamente 4,8 vezes maior após o programa de capacitação^(27)^.

Neste estudo, a taxa de expulsão foi de 2%, demonstrando uma perspectiva positiva sobre a adaptação do método. Esses dados coincidem com achados internacionais na Austrália, onde, de 207 inserções acompanhadas pelo profissional capacitador, 91% das inserções foram bem-sucedidas e não contaram com a supervisão direta^(28)^.

É notável que as localidades que implantarem ou ampliarem os serviços de planejamento familiar/reprodutor estarão contribuindo para o empoderamento feminino e o acesso ao serviço, que colabora com a redução da mortalidade materna^(5,11,29)^.

Os participantes compartilharam as experiências utilizadas para sensibilizar as mulheres na adesão ao DIU, como a divulgação por agentes comunitários de saúde, de modo a evidenciar o trabalho multiprofissional. Nesse sentido, é importante promover a capacitação de todos os trabalhadores envolvidos para a melhor produção do trabalho em saúde^(29)^.

Os dados registrados no SISAB abrangeram a assistência em saúde reprodutiva, e chama a atenção o total de mulheres que receberam DIU nos 12 municípios onde os participantes atuam, em contraposição às demais 67 cidades do estado do Mato Grosso do Sul. Esta informação faz refletir se todos os profissionais realizam os devidos registros dos atendimentos, ação de grande importância para mensurar indicadores da meta 3.7 dos ODS^(5)^.

Os resultados somam-se a outros estudos^(11,14,28)^, que evidenciaram a ampliação do acesso ao DIU por meio do aumento da força de trabalho em saúde sexual e reprodutiva e o potencial de redução da mortalidade materna relacionada à gravidez não planejada e não desejada.

### Limitações do estudo

Como limitações, considera-se a amostra por conveniência, na qual participaram enfermeiros que foram habilitados pelo Coren na gestão 2018 a 2023, não sendo representativo de todo o estado de Mato Grosso do Sul. Contudo, os resultados obtidos são semelhantes aos de outras pesquisas nacionais^(11,14,28)^, que também evidenciaram a ampliação do acesso ao DIU por enfermeiros da APS, indicando possibilidades que podem ser reproduzidas em outras localidades para o investimento na habilitação de enfermeiros no manejo dos LARCs.

### Contribuições para as áreas de enfermagem, saúde ou políticas públicas

A habilitação de enfermeiros para o manejo do DIU por enfermeiros representa recursos humanos disponíveis nas ações que contribuem para a redução da gravidez não desejada, aborto e mortalidade marterna. Fomentou o compromisso nacional junto à ONU de, até 2030, assegurar o acesso universal aos serviços e insumos de saúde sexual e reprodutiva, incluindo o planejamento reprodutivo, acesso à informação e educação, bem como a integração da saúde reprodutiva em estratégias e programas nacionais.

## CONCLUSÃO

Habilitar enfermeiros para o manejo dos métodos contraceptivos de longa duração é ação estratégica para ampliar a força de trabalho em prol da saúde sexual e reprodutiva, especialmente pelo SUS. Ademais, o estudo se equipara a outros estudos nacionais e internacionais, dispondo de taxas semelhantes, evidenciando competência para o manejo adequado do DIU.

Cabe aos gestores a implementação das políticas públicas de saúde para, com isso, prover as estruturas necessárias para um processo de trabalho seguro, eficaz e em conformidade com as evidências científicas e legais.

Este estudo configura-se como uma inovação às práticas de enfermagem brasileira, pois revelou ser possível melhorar a saúde reprodutiva e, com isso, reduzir as gestações não planejadas e suas consequências.
